# Real-world experience with the use of diazoxide among people living with congenital hyperinsulinism and their caregivers

**DOI:** 10.3389/fendo.2025.1628125

**Published:** 2025-08-21

**Authors:** Tai L. S. Pasquini, Kristen E. Rohli, Fiona J. Almeida, Indraneel Banerjee, Antonia Dastamani, Diva D. De Leon, Lauren N. Lopez, Paul S. Thornton, Julie Raskin

**Affiliations:** ^1^ Congenital Hyperinsulinism International, Glen Ridge, NJ, United States; ^2^ Department of Health Promotion and Policy, School of Public Health & Health Sciences, University of Massachusetts Amherst, Amherst, MA, United States; ^3^ Department of Paediatric Endocrinology, Royal Manchester Children’s Hospital, Manchester, United Kingdom; ^4^ Department of Paediatric Endocrinology and Diabetes, Great Ormond Street Hospital for Children, National Health Service (NHS) Foundation Trust, London, United Kingdom; ^5^ Congenital Hyperinsulinism Center and Division of Endocrinology and Diabetes, Department of Pediatrics, Children’s Hospital of Philadelphia, Perelman School of Medicine, University of Pennsylvania, Philadelphia, PA, United States; ^6^ Hyperinsulinism Center, Division of Endocrinology, Cook Children’s Medical Center, Fort Worth, TX, United States

**Keywords:** congenital hyperinsulinism, hypoglycemia, diazoxide, rare diseases, endocrinology, natural history, patient-reported data, registry

## Abstract

**Introduction:**

Congenital hyperinsulinism (HI) is a rare disease that causes severe hypoglycemia. Diazoxide is the first-line treatment; however, many individuals using diazoxide continue to experience hypoglycemia. Diazoxide is associated with side effects that impact life and well-being.

**Methods:**

The study utilized a mixed-methods approach combining structured, survey-based cross-sectional quantitative data from the HI Global Registry (HIGR) (n=165, 89% were caregivers), of whom 75% reported current diazoxide use, with qualitative interviews with caregivers (n=12) and individuals with HI (n=6). This is the first mixed-methods study to focus on the experience of diazoxide treatment as reported by the individual taking the medicine and/or their caregiver.

**Results:**

Of HIGR participants, 93% reported at least one side effect, including hypertrichosis (89%), loss of appetite (40%), facial changes (23%), and swelling (22%) with diazoxide use. In HIGR, 37% of people currently on diazoxide reported experiencing hypoglycemia up to several times per week. Interview participants described how these side effects, the drug’s taste, and feeding difficulties associated with HI and diazoxide adversely impacted daily life.

**Discussion:**

Diazoxide is commonly used by families living with HI, but a significant proportion reported hypoglycemia. Individuals who experienced better glycemic control with the drug were less critical of side effects. Combining HIGR data with in-depth interviews facilitated understanding of day-to-day life, which can help implement measures to better support families managing HI. This study prompts the need for improved treatment options and for clinicians to utilize the International HI Care Guideline to optimize diazoxide therapy.

## 1 Introduction

Congenital hyperinsulinism (HI) is a rare disease characterized by excessive insulin secretion that leads to severe hypoglycemia. HI occurs in approximately 1 in 28,000 births in most countries, and is the most frequent cause of prolonged hypoglycemia in newborns and children (1, 2). Early diagnosis and effective management of this disease are necessary to avoid prolonged hypoglycemia, which is associated with an elevated risk of brain damage, seizures, developmental delay, and death (3, 4). HI usually presents in the newborn period (5, 6). A diagnosis of HI relies on the demonstration of a biochemical profile consistent with inappropriate secretion/actions of insulin during a spontaneous or provoked episode of hypoglycemia, including detectable plasma insulin/C-peptide, low plasma levels of free fatty acids and beta-hydroxybutyrate, and a glycemic response to a pharmacologic dose of glucagon (7, 8). Management of HI involves vigilant glucose monitoring and can include medications, surgery, and/or feeding plans to minimize, and optimally prevent, hypoglycemia (8, 9).

Diazoxide is the first-line treatment for HI, and is the only medication for HI approved by the United States Food and Drug Administration and European regulatory authorities (8). Diazoxide blunts insulin secretion by activating ATP-sensitive potassium (K_ATP_) channels and hyperpolarizing the β-cell plasma membrane (10, 11). An individual is considered responsive to diazoxide by demonstrating a reversal in hypoketotic hypoglycemia (8). Up to 60% of individuals with HI are unresponsive to diazoxide (12–17); unresponsiveness often results from K_ATP_ channel defects, secondary to inactivating pathogenic variants in *ABCC8* and *KCNJ11*, the genes encoding the channels, although other forms of genetic HI may also be diazoxide-unresponsive (12). Responsiveness is not universally assessed according to the standards described in the international HI care guidelines, which may be a contributing factor as to why some individuals on diazoxide may continue to experience hypoglycemia. Data from the HI Global Registry (HIGR) have shown that up to 32% of people currently taking diazoxide report hypoglycemia (blood glucose level below 70 mg/dL) multiple times a week or more frequently (6, 18).

Several studies have reported that side effects accompany diazoxide treatment (13, 19, 20). It is estimated that up to 9.7% of individuals discontinue use due to serious side effects impacting the cardiovascular/respiratory, hematologic, or gastrointestinal system (21). Individuals with perinatal stress-induced hyperinsulinism have higher rates of serious side effects (8, 16, 21, 22). The most frequently reported side effect of diazoxide is hypertrichosis, impacting up to 84% of people taking diazoxide (6). Changes in facial features are reported in up to 24% of people (9, 19, 23). Fluid accumulation, pulmonary hypertension, and pulmonary overload, which may become life-threatening, have also been observed (21, 24–27). The use of a diuretic to mitigate fluid retention is the current standard of care on initiation of diazoxide (8, 19). Other reported side effects include cardiac failure (28, 29), neutropenia, thrombocytopenia, and hyperuricemia (21, 25, 30). To monitor the occurrence of side effects, clinicians are advised to perform periodic echocardiograms, complete blood counts, and serum uric acid analysis on individuals who are prescribed diazoxide (8, 9, 27).

Multiple reports suggest an increase in feeding difficulty after diazoxide initiation (22, 31). Feeding difficulties are also a feature of HI; therefore, it is difficult to determine whether this is a feature of the disease or the treatment (18, 32). Moreover, gastrointestinal reactions have been reported in individuals receiving diazoxide, which may further exacerbate challenges in encouraging nutritional intake, which is critical to maintaining euglycemia (21, 33–35). Some diazoxide users were also tube fed; tube feeding may be required to ensure swift and easy access to nutritional support to maintain glycemic control (31).

In previous publications that have included the patient voice, there was some evidence that caregivers and individuals with HI taking diazoxide experienced a psychosocial burden due to the impact of the visible side effects, such as hair growth, which may trigger social distress and stigmatization for families (18, 32). Despite the acknowledgment of diazoxide’s side effects by clinicians and families affected by HI, the impact of these side effects is not adequately reported in the literature. There is a growing interest in including patient-reported data and the impact of treatments on a patient’s quality of life in research and clinical studies, often in the context of regulatory decision-making. However, this work has often not been conducted for treatments already on the market. Prior to this study, no study specifically asked caregivers and patients with HI about their experiences with diazoxide. The aim of this research was to better understand the real-world impact of diazoxide and its side effects on people living with HI and on their caregivers through their own words, including the impact on glycemic control, daily routines, and quality of life.

## 2 Methods

A mixed-methods approach utilized structured quantitative data from HIGR (n= 165, 89% were caregivers) and qualitative interviews with caregivers (n=12) and individuals living with HI (n=6). The study was approved by North Star Review Board (NB400184). HIGR data on side effects and glycemic control were summarized to inform the qualitative interviews, which expanded on the study’s key themes. The HIGR data was utilized to capture the retrospective experience for a large group of people, while the interviews provided direct and focused insight into their lived experience.

### 2.1 HI global registry

HIGR was launched in October 2018 as the first global patient-powered HI registry (6). The registry is available in eight languages and contains 13 patient and/or caregiver surveys that collect information through multiple-choice and open-ended questions on the experience of HI over a person’s lifetime and one physician-reported survey (6). HIGR recruitment is ongoing through Congenital Hyperinsulinism International’s (CHI) virtual platforms and in-person at patient-family conferences and HI clinics. Participation is open to individuals over the age of 18 who have received a diagnosis of HI and caregivers of children (younger than 18 years) with HI who are reporting on their child’s experiences and the impact on their own quality of life.

HIGR surveys were created by an international team of HI experts, including family members of children with HI, clinicians, and researchers, based on the literature and lived experiences (6). No tool has yet been validated for HI research; however, the HIGR quality of life surveys were based on validated tools, including PedsQL and DisabKids (37, 38). The surveys are cross-sectional and largely retrospective. A few surveys, including quality of life and glucose monitoring, are longitudinal. In this study, we included responses from the most recent survey submissions.

The authors reviewed the responses from the HIGR surveys relating to medication management, other medical conditions, diet and feeding, patient quality of life, caregiver quality of life, and glucose monitoring, to inform the qualitative interview script. After completing the interviews, the authors generated a data report for all HIGR participants who had completed the medication management survey by September 2024. Survey questions included in this report may be made available upon reasonable request.

Data was analyzed based on current or previous diazoxide use (minimum four weeks). Individuals could choose to answer individual questions and surveys, resulting in a variable number of participants (n) for each question. The total number of people who answered the question is provided for each data point. The participant’s age is reported as that at survey completion. Data were summarized and visualized using Posit PBC RStudio (version 2024.04.2 + 764).

### 2.2 Qualitative methodology: interviews

Participants were eligible if they signed an informed consent form, were willing to participate in a one-time 60-minute web-based interview, could speak English, and had completed all HIGR surveys. Participants were eligible if they or their child was currently taking diazoxide (any length of use) or had previously used the drug for an extended period (minimum 10 years). Interview participants included caregivers, all mothers, and adults with HI aged 18 years or older. Children were invited to participate in a 30-minute interview if a parent was present and the child provided written assent. Recruitment channels included CHI’s website, social media, and email list.

A total of 50 people expressed interest in participating in the study. Of this group, 26 were identified as eligible to participate, and 22 people were invited to participate; these 22 people represented a range of ages and primary treatment centers. Four did not respond to requests to schedule interviews prior to data saturation for the study being met. In total, 18 people participated in the qualitative interviews. Of this group, 12 were caregivers of individuals living with HI, three were children aged 10–12 years, and three were young adults living with HI (**Table 1**).

**Table 1:** Characteristics of the qualitative interview study population.

Participant identifier	Age (years)	Sex	Race	Age started diazoxide	Diazoxide use
1CG	1	M	More than one race	3 weeks	Current
2CG	7	F	White	2 weeks	Current
3CG 3HI	12	M	White	2 weeks	Previously used for 10–14 years
4CG 4HI	10	F	Unknown	1 week	Current
5HI	22	M	White	4–6 months	Previously used for 10–14 years
6HI	26	F	White	3 years*	Current
7CG	2	F	White	7 months	Current
8CG	4	M	More than one race	11 months	Current
9CG	2	F	White	6 weeks	Current
10HI	26	F	White	2 years	Current
11CG	6	F	White	9 months	Current
12CG	8	M	White	16 months	Current
13CG	2	F	More than one race	2 weeks	Current
14CG 14HI	10	F	White	3 months	Current
15CG	5	F	More than one race	3 months	Current

CG and HI in the participant identifier refer to caregiver and person with HI, respectively. F, female; M, male.

*Note this participant discontinued use for a number of years in her childhood before restarting diazoxide four years ago.

Interviews were conducted during March and April 2024. Caregivers and adults who participated in the interview received a $125 gift card, and the parents of the children who participated were given an additional $75 for their time.

Interview scripts were developed with open-ended questions, and follow-up questions were used for additional exploration of themes. The interviews focused on the participants’ experiences, feelings, and perceptions of diazoxide and its side effects; additional questions related to interactions with healthcare professionals, glycemic control, and quality of life. Interview questions were developed in conjunction with a psychologist and pediatric endocrinologists with expertise in HI. All interviews with adults were conducted by the same author on a one-to-one basis, whereas in the interviews with children, one of the interviewee’s parents was also present.

Qualitative data analysis combined Van Manen and grounded theory strategies, including immersive reading, open coding, thematic identification, interpretative analysis, and integration to create a comprehensive narrative (39–41). Data analysis was completed manually by two authors using Delve, a qualitative data analysis software program. Open coding focused on identifying keywords or phrases, which were then contextualized through axial and selective coding to generate codes and subcodes to create the final codebook. Peer debriefing was completed throughout coding with individuals who were familiar with diazoxide as well as with those who were not, to ensure methodological consistency, clarity of interpretation of the data, and reproducibility. Interview participants were assigned a participant identifier to ensure anonymity, and basic demographic data were recorded (**Table 1**). Interview participants lived in Australia, Canada, Italy, the United Kingdom, the United States, and the Caribbean. Based on the risk of identifiability, the specific country in the Caribbean has not been shared, and the countries of residence were not recorded in **Table 1**.

Quotes were edited to improve clarity without changing their meaning (e.g., removing “like”). Quotes have been anonymized, but were attributed to the participant via their identifier, which includes a letter code specifying whether the participant is a caregiver (CG) or a person with HI (HI), and the age of the person with HI. An example of the qualitative codebook and the themes that emerged are available in **Supplementary Table 1**.

### 2.3 Participants

In total, 165 HIGR participants reported either current use (n = 123) or past use (n = 42) of diazoxide (**Table 2**). Throughout the manuscript, we will refer to individuals who were currently taking diazoxide at the time of the study; this does not indicate current use today. In HIGR, about one-third of individuals who were currently taking diazoxide were aged 1–3 years (34%); of these, 62% were from North America (of whom 88% were from the United States) and 59% were White. All individuals in HIGR taking diazoxide and all the interview participants indicated diffuse or atypical forms of HI. The genetic background of the HIGR participants varied. K_ATP_ channel variants were present in 22% of individuals currently taking diazoxide and 45% of the past use group. In the current use group, 32% of participants received negative genetic testing results, and 22% either had no genetic testing or did not report genetic testing. For individuals in HIGR, 29% had taken diazoxide for 6 years or longer (**Table 2**). The patients’ ages ranged from 1 year to 26 years for interview participants. Five of these individuals began diazoxide within the first month of life, and seven of them within the first year of life (**Table 1**). Two people with HI were previous diazoxide users.

**Table 2:** Characteristics of the HIGR survey study population.

	Current use (n = 123)	Past use (n = 42)	Total (n = 165)
Sex, n (%)			
Male	66 (54)	23 (55)	88 (54)
Female	57 (46)	19 (45)	76 (46)
Age, n (%)			
< 1 year	19 (15)	3 (7)	22 (13)
1–3 years	42 (34)	10 (24)	52 (32)
4–6 years	20 (16)	9 (21)	29 (18)
7–9 years	12 (10)	4 (10)	16 (10)
10–13 years	15 (12)	6 (14)	21 (13)
14–17 years	3 (2)	1 (2)	4 (2)
≥ 18 years	12 (10)	9 (21)	21 (13)
Continent of residence, n (%)			
Africa	1 (1)	0 (0)	1 (1)
Asia	3 (2)	3 (7)	6 (4)
Europe	30 (24)	8 (19)	38 (23)
North America	76 (62)	26 (62)	102 (62)
Oceania	8 (7)	2 (5)	10 (6)
South America	5 (4)	3 (7)	8 (5)
Race, n (%)			
American Indian or Alaska Native	1 (1)	1 (2)	2 (1)
Asian	1 (1)	0 (0)	1 (1)
Black or African American	1 (1)	0 (0)	1 (1)
White	73 (59)	24 (57)	97 (59)
More than one race	10 (8)	2 (5)	12 (7)
Unknown or not reported	37 (30)	15 (36)	52 (32)
HI type, n (%)			
Diffuse	64 (52)	21 (50)	85 (52)
Focal	0 (0)	5 (12)	6 (4)
Atypical, other, unknown	58 (48)	16 (38)	74 (45)
Genetic testing results, n (%)			
Non-K_ATP_ channel variant	30 (24)	7 (17)	37 (22)
K_ATP_ channel variant	27 (22)	19 (45)	46 (28)
Negative genetic testing	39 (32)	8 (19)	47 (28)
No genetic testing performed	23 (19)	6 (14)	29 (18)
Not reported	4 (3)	2 (5)	6 (4)
Length of diazoxide use, n (%)			
< 6 months	25 (20)	19 (45)	44 (27)
6–12 months	20 (16)	2 (5)	22 (13)
1–5 years	37 (30)	10 (24)	47 (28)
6–10 years	20 (16)	2 (5)	22 (13)
> 10 years	18 (15)	9 (21)	27 (16)
Unknown	3 (2)	0 (0)	3 (2)

Among the 83 participants who responded to whether they identify with a community known to have a higher incidence of HI, 10% of individuals currently using diazoxide and 15% of past users belong to at least one, including Ashkenazi Jewish and Turkish communities. In addition, of 163 total individuals, 5% of those currently using diazoxide and 5% of past users reported having a syndrome known to more frequently occur with HI, including Beckwith-Wiedemann syndrome, Kabuki syndrome, Fanconi syndrome, polycystic kidney disease, and Rubinstein–Taybi syndrome. HI, congenital hyperinsulinism; HIGR, HI Global Registry; K_ATP,_ adenosine triphosphate-sensitive potassium channel.

## 3 Results

Qualitative interviews identified transversal themes overlapping topic areas, with participants not being able to attribute a symptom or experience definitively to HI versus the drug. Of HIGR participants, 93% reported at least one side effect. However, in the interviews, topics related to feeding issues and the difficulty of managing their daily life were more challenging to attribute exclusively to the drug as opposed to a feature of HI. Demonstrative quotes in the text and figures highlighted the major codes identified through qualitative data analysis and provided depth to the results of the HIGR survey data.

### 3.1 Diazoxide administration

Diazoxide is available as a liquid/oral suspension or in tablet/capsule form but is typically available in one form in each country. In HIGR, of the 123 participants currently taking diazoxide, 80% receive a liquid/oral suspension. HIGR survey participants who were currently taking diazoxide (n = 123) reported different frequencies of medication intake, specifically, once a day (5%), twice a day (59%), or three times a day (36%). Interview participants reported less disruption when they took the medicine once or twice a day compared with three times a day because they could schedule dosing before and/or after school.

**Figure 1:**
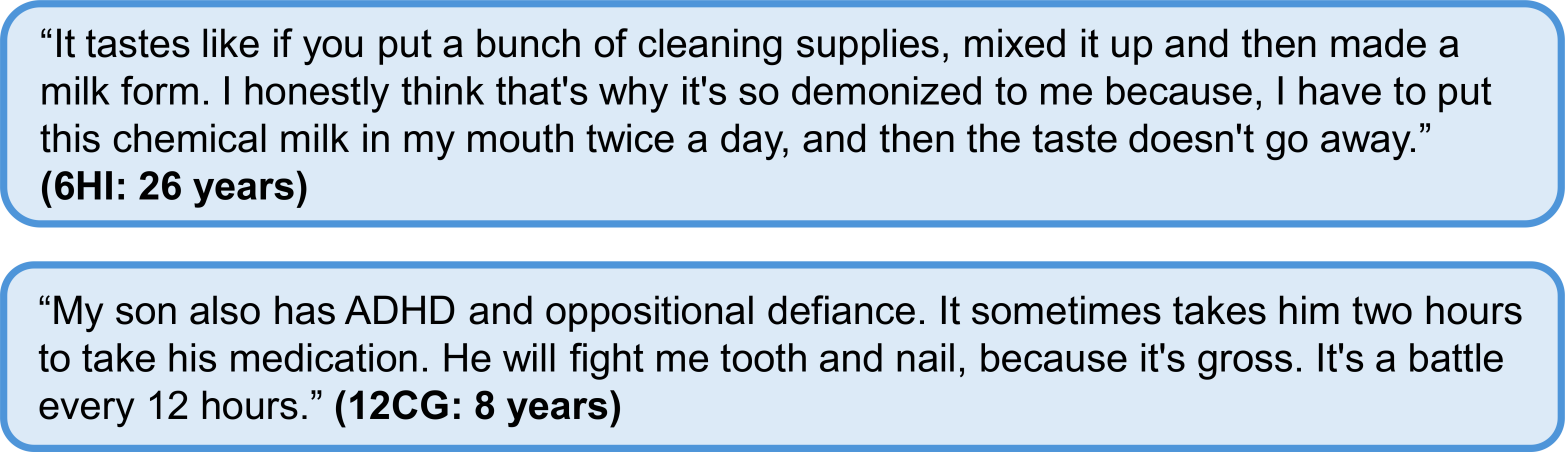
Additional quotes relating to diazoxide administration. Participant identifier and age are given in parentheses. In the participant identifier, CG and HI refer to caregiver and person with HI, respectively. ADHD, attention deficit hyperactivity disorder; HI, congenital hyperinsulinism.

Taste of diazoxide was frequently commented upon, with patients and caregivers describing this medicine as tasting like “chemical milk,” “charcoal with licorice,” or “very dirty water,” and using words like “bitter,” “sour,” “gross,” and “disgusting” (**Figure 1**). The taste contributed to additional challenges, leading to families developing strategies to mitigate the residual taste.

Some individuals discussed having an immediate gastrointestinal reaction to diazoxide.

The diazoxide made her immediately throw up. She didn’t like it, but we could coax her into taking it, but it went into her stomach, and it just came back, right back out. (11CG: 6 years)

One parent whose child did not have a problem with the taste or taking the medication theorized that their child was compliant because he had been taking the drug since he was a baby. Another individual whose child was using a gastrostomy tube for additional carbohydrates, stated that the feeding tube was also used to administer the medication, eliminating the need for oral administration, thereby bypassing taste concerns and associated fights. Parents of older children discussed their child’s maturity or desire to be independent as a reason why they no longer experienced resistance to taking diazoxide.

Despite expressed challenges in taking diazoxide, adherence was high among families with younger children. Participants reported that tools such as alarms were used to ensure a dose was not missed. Only three parents could identify instances in which their child missed a dose, and they could detail the circumstances of the two to three missed doses. However, parents with older children had concerns about adherence because the responsibility for taking diazoxide was shifting from them to their child.

Only one adult with HI said that they discontinued diazoxide on a vacation, due to lack of access and to test the effect of discontinuing the medication, but they resumed diazoxide use two months later. Only one family spoke about having problems accessing the drug based on geographic location. Of individuals currently taking diazoxide in HIGR (n = 121), 14% stated that they had issues accessing the drug. While most people interviewed were able to obtain diazoxide, participants reported a fear of shortages and disruptions due to their experiences with supply chain issues. To ensure a constant supply, one participant had to drive hours to get the drug, others stored excess supply as backup, and another required medical professionals to intervene on the family’s behalf.

### 3.2 The impact of HI and diazoxide on diet and gastrointestinal health

**Figure 2:**
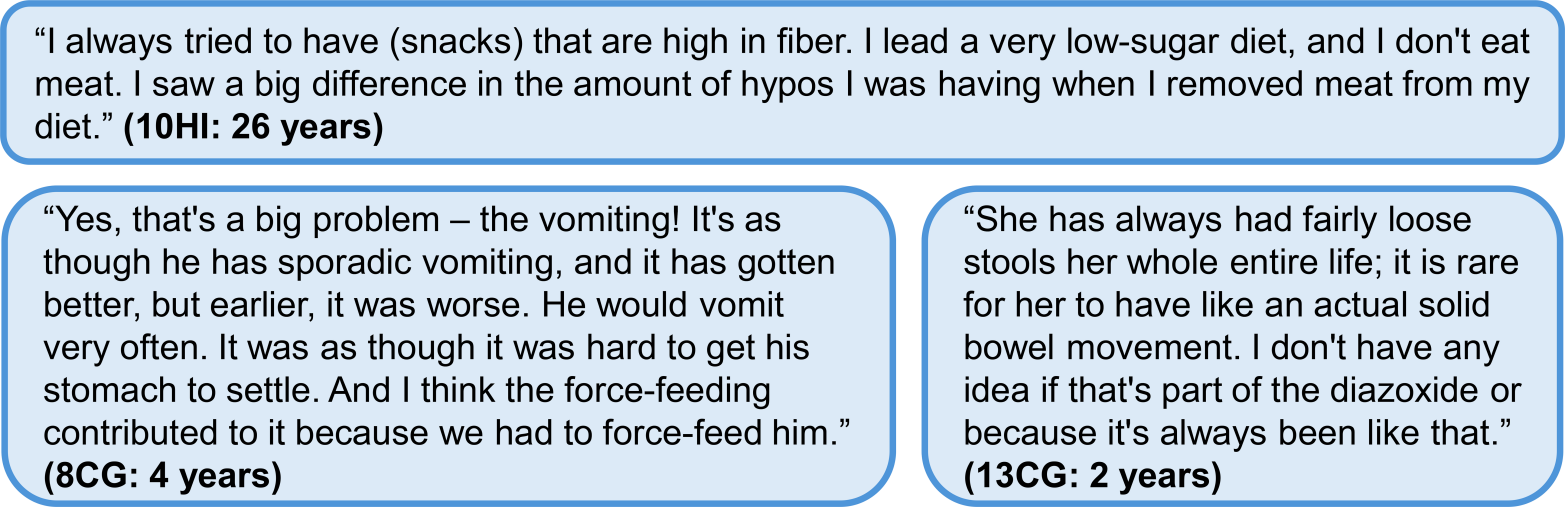
Additional quotes relating to the impact of diazoxide and HI on diet and gastrointestinal health. Participant identifier and age are given in parentheses. In the participant identifier, CG and HI refer to caregiver and person with HI, respectively. GI, gastrointestinal; HI, congenital hyperinsulinism.

The complex relationship between HI, diazoxide, feeding issues, and gastrointestinal problems emerged as a prominent theme in the interviews. This finding correlated with HIGR participants currently taking diazoxide (n = 111), of whom 64% reported feeding issues (**Table 3**), and 28% reported five or more feeding issues on a regular basis. The most common feeding issues experienced included poor appetite (29%), refusing to eat (19%), and gagging (13%). However, 22% of these 111 individuals had experienced resolution of all feeding issues, suggesting temporal improvement. Of those participants currently on diazoxide with resolved feeding issues (n = 24), 83% reported that the feeding issues were resolved by age 7. The most common feeding issues discussed in the interviews were poor appetite and refusal to eat (**Figure 2**).

**Table 3:** Feeding experiences reported by HIGR participants currently taking diazoxide.

	Participants		
Has the participant experienced any feeding issues on a regular basis? n (%)	n = 111		
No	40 (36)		
Yes	71 (64)		
Of those that reported feeding issues	n = 71		
Currently experiencing feeding issues, n (%)	44 (62)		
All feeding issues resolved, n (%)	24 (34)		
Did not report current feeding issue status, n (%)	3 (4)		
Feeding issue experienced, n (%)^a^	n = 111		
	Ever		Currently
Poor appetite	51 (46)		32 (29)
Refusing to eat	43 (39)		21 (19)
Reflux	34 (31)		9 (8)
Vomiting	32 (29)		11 (10)
Gagging	31 (28)		14 (13)
Slow eating	30 (27)		13 (12)
Problems with texture	23 (21)		12 (11)
Overeating	12 (11)		4 (4)
Coughing	20 (18)		9 (8)
Uncoordinated oral skills	19 (17)		9 (8)
Other	4 (4)		2 (2)
Has the participant used tube feeding? n (%)	n = 112		
No	65 (58)		
Yes (past or present)	47 (42)		
Type(s) of tube feeding used, n (%)^a^	n = 47		
	Currently	In the past	
Tube feeding: NG or OG tube^b^	2 (4)	38 (81)	
Tube feeding: G-tube, G-button, J-tube, or PEG	9 (19)	7 (15)	
TPN (long-term intravenous feeding)	0 (0)	2 (4)	

G-button, gastrostomy button; G-tube, gastrostomy tube; HIGR, HI Global Registry; J-tube, jejunostomy tube; NG, nasogastric; OG, orogastric; PEG, percutaneous endoscopic gastrostomy; TPN, total parenteral nutrition.

a Participants could select more than one response as appropriate; therefore, the total proportion will not equal 100%. Additionally, individuals could choose to answer individual questions and surveys, resulting in a variable number of participants (n) for each question.

b One participant reported using NG or OG tube feeding but did not specify current or past use.

We don’t know if it is because of the side effect of the medication that she could lose her appetite or is not that hungry, or because she goes low (during the) daytime … Maybe she’s just not that hungry because we snacked her too much. Quite tricky to know. (15CG: 5 years)

I was just not hungry and I think it’s because diazoxide also gives you that nauseous taste in your mouth, which definitely inhibits things. (6HI: 26 years)

Diet was a central consideration for everyone in the qualitative study, who were focused on what foods they ate and when. Multiple caregivers stated that meal planning revolved around their child with HI to maintain euglycemia.

We cater to her because it makes our life easier, [then] we don’t have to take her to a hospital. If she wants SpaghettiOs, that’s what she’s getting. If she wants pizza, that’s what she’s getting. Because she’ll eat it and she’ll eat it well, and her blood sugars will be good. (11CG: 6 years)

During the interviews, eight people discussed the importance of a balance between carbohydrates, dairy, protein, fruits, and vegetables. Importantly, the order in which people ate these different foods and the amounts eaten were precisely described. Three individuals in the study indicated that they had a protein sensitivity. Overall, complex carbohydrates were particularly important in the diet to prevent severe low blood glucose.

Besides the diazoxide, we manage her disease with food, especially with complex carbs … We saw that even with the same dose every day, if she changed the dose of a complex carb, the glucose drops and so it’s a really important part of our management for this disease. (7CG: 2 years)

Ensuring that the person with HI ate enough before bedtime was mentioned by four interviewees. HIGR survey participants currently taking diazoxide (n=109) were asked about the length of fasting tolerance before their blood glucose level dropped below 70 mg/dL (3.9 mmol/L, 0.7 g/L): responses showed that 53% of the 109 people with HI could last 4 hours or less during daytime and 36% could not go more than 8 hours overnight. Pre-bedtime snacks were often discussed as part of the routine (**Figure 2**).

We do some modifications to diet to try and help keep blood sugar controlled. Whether or not they’re actually necessary, is sort of TBD (to be determined). But we do always make sure that she has a fairly healthy carbohydrate load before bed and that she is having snacks throughout the day. (13CG: 2 years)

For individuals with HI, tube feeding is often used to maintain blood glucose levels; for some, it is used to support nutritional needs resulting from feeding issues. Of HIGR participants currently using diazoxide (n = 112), 42% have used tube feeding, including 10% who utilized tube feeding at the time of the study. Tube feeding methods included one or more of the following: the use of a nasogastric or orogastric tube, a gastrostomy tube or button, a jejunostomy tube, and/or total parenteral nutrition (**Table 3**). One individual in the qualitative study was currently using tube feeding, and three others had used tube feeding at some point.

She does get low, and because we’re feeding her every 30 minutes, not as often, but if she’s running hard or playing hard … you feel really bad just being like, sorry, I have to hook you up to a feeding pump. Now I have to stop you from enjoying yourself. She still does sometimes go low. But we feed so often that I think that prevents it more than just the medication. (9CG: 2 years)

Among HIGR survey participants who were currently using diazoxide and who reported on their comorbid conditions (n = 81), 73% reported having at least one other diagnosed medical condition (**Table 4**). The most frequently reported comorbidities included attention deficit hyperactivity disorder (28%), epilepsy (22%), and autism spectrum disorder (20%). Of the participants with no comorbidities at the time of the survey (n = 22), 64% were 6 years or younger, suggesting that a comorbid condition may not yet have been identified.

**Table 4:** Comorbid conditions reported by HIGR participants currently taking diazoxide.

Has the participant been diagnosed with any other medical condition in addition to HI? (n = 81)	n (%)
No	22 (27)
Yes	59 (73)
Medical condition (n = 80)^a^	n (%)^b^
No medical condition	22 (37)
Anxiety disorder	7 (12)
Asthma	7 (12)
Attention deficit hyperactivity disorder	17 (28)
Autism spectrum disorder	12 (20)
Cerebral palsy	5 (8)
Epilepsy	13 (22)
GE reflux or GERD	11 (18)
Heart-related condition	11 (18)
Learning disability (in reading, written expression, mathematics, or other specified activity)	10 (17)
Language disorder or auditory processing disorder	4 (7)
Kidney-related condition	3 (5)
Migraine or headache disorder	3 (5)
Strabismus	3 (5)
Other^c^	12 (20)

GE, gastroesophageal; GERD, gastroesophageal reflux disease; HI, congenital hyperinsulinism; HIGR, HI Global Registry.

a Of the 81 participants, 1 indicated having another medical condition but did not specify which condition.

b Participants could select more than one medical condition, if appropriate; therefore, the total proportion will not equal 100%.

c ’Other’ indicates conditions reported by fewer than three participants: blood-related condition (not including blood cancer) (n = 1), depressive disorder (n = 1), gastrointestinal motility disorder (n = 2), hearing impairment or deafness (n = 1), inflammatory bowel disease (n = 1), intellectual disability (n = 2), menstrual-related condition (n = 2), obsessive-compulsive disorder (n = 2), and scoliosis (n = 2).

Of those current diazoxide users in HIGR who also reported on their diagnosed comorbid conditions (n = 59), gastrointestinal comorbidities were reported by 22% (**Table 4**) and included gastroesophageal reflux, gastrointestinal motility disorder, and inflammatory bowel disease. No respondent specified whether these conditions were explicitly linked to their HI or diazoxide use. In the qualitative interviews, gastrointestinal issues ranged from minor to severe, including one person who received a diagnosis from a neurologist of abdominal migraines. Multiple caregivers described their child as being “a spitty baby” or having “reflux issues”. Vomiting was discussed as a common problem associated with taking diazoxide (**Figure 2**).

I would say she still vomits probably every day, at least once. She also just has a very sensitive gag reflex, I think from all the vomiting … Originally, if (food) just got stuck, she would just vomit, it would just come straight back out, and that was almost every time she tried to eat. (11CG: 6 years)

Other bowel-related issues were also described, including constipation, bloating, and diarrhea. A few individuals also discussed the consistency of their child’s stool, using descriptions such as “sticky” and “very loose.”

She’s always had interesting stomach issues, which we’ve investigated over the years. We can’t tell exactly what is diazoxide and what is something else. But you know, there are all those things that get checked on the boxes of side effects for diazoxide that it possibly could be that. She has always been a good eater, yet we have had different unexplained stomach issues and gastro issues over the years that no matter how much looking, no one’s been able to really put a finger on anything else that could cause it. (4CG: 10 years)

Feeding issues adversely impacted blood glucose levels with the potential for hospitalization. Of HIGR participants currently taking diazoxide (n = 119), 35% reported one or more hospitalizations within the last year due to problems related to blood glucose.

When she gets a stomach, like a throw up thing, that’s the biggest. We’ve been hospitalized a couple of times for that … We try to make sure she gets her dose and if she can keep it down, then I’m like, “she’s good”. But if she doesn’t, that’s what kind of freaks me out. And I usually call and we’ll up her dose automatically. (14CG: 10 years)

The direct role of diazoxide in feeding challenges was unclear. The effort necessary to achieve nutrition-supported glycemic control was consistent across the conversations, revealing a complex relationship with food among diazoxide users. One person mentioned that they had a diagnosed eating disorder that required treatment.

### 3.3 Side Effects

Of participants in HIGR who had ever taken diazoxide (n = 160), 94% reported at least one side effect while taking the drug; of these, at least 15% reported that they stopped taking the drug, temporarily or permanently, owing to the side effects they experienced. Of individuals currently taking diazoxide who experienced side effects (n = 113), 49% reported having experienced at least three, and one person experienced 11 side effects. The frequency of side effects experienced by HIGR participants is listed in **Table 5**. All individuals in the interviews indicated that they or their child experienced some side effects associated with diazoxide, using phrases like “pretty nasty” and “gnarly” to describe them.

**Table 5:** Side effects experienced by HIGR participants currently taking diazoxide.

Side effect, n (%)^a^	Participants currently taking diazoxide (n = 121)
No side effects	8 (7)
Increase in growth of body hair	108 (89)
Loss of appetite	48 (40)
Facial changes	28 (23)
Swelling (hands, feet, or both)	27 (22)
Stomach pain or upset	26 (21)
Hyperglycemia	14 (12)
Skin rash	13 (11)
Racing heartbeat (tachycardia)	11 (9)
Changes in sense of taste	10 (8)
Headache	6 (5)
Pulmonary hypertension	6 (5)
Dizziness	5 (4)
Fluid in the lungs	4 (3)
Other^b^	10 (8)

a Participants could select more than one side effect, if appropriate; therefore, the total proportion will not equal 100%.

b Selecting ‘other’ allowed a free-text response. Responses were as follows: thrombocytopenia (n = 2), sadness and hair loss (n = 1), apnea and cardiac arrest (n = 1), constipation (n = 1), neutropenia (n = 1), muscle twitching in hands, legs, and face (n = 1), facial swelling (n = 2), and scrotum swelling (n = 1).

#### 3.3.1 Side effect: hair

An increase in body hair growth was reported as a side effect for 89% of the 121 current diazoxide users in HIGR (**Table 5**), and the effect on hair was described in all but one interview. This included both positive and negative descriptions of “excess hair,” “hair darkening,” “hair growth,” and “nice hair”; three parents said that their child looked like a monkey. Hair growth was commonly described as occurring all over the body and specifically cited as growing on the back, forehead, legs, arms, hands, upper lip, eyebrows, mustache, and pubic area.

When he was probably three or four years old his arms were covered in hair. He had hair on his forehead. He had hair on his legs. He had hair on his back, he had hair on his butt. He literally almost looked like a monkey. (3CG: 12 years)

Participants expressed concern about the reactions of other people, which often guided their decisions about hair management. Others described frequent haircuts, shaving, or bleaching, and one adult said she had undergone laser hair removal.

He’s in third grade, kids notice now, they’re pretty ruthless … They cut the forehead, and it grows all the way up through his neck. He says kids are noticing he has a mustache already. (12CG: 8 years)

Some parents indicated that hair management was less cosmetic and more practical.

We just started shaving her arm where she wears her Dexcom just because it’s painful to take it off, so that’s the first time we’ve ever shaved her. (13CG: 2 years)

We used to clip with hair clippers the hair on certain parts, just because she would get so hot or, in diapers, nappies and stuff like that. The hair on the back would get all caught up with all sorts of stuff. (4CG: 10 years)

#### 3.3.2 Side effect: swelling and fluid retention

**Figure 3:**
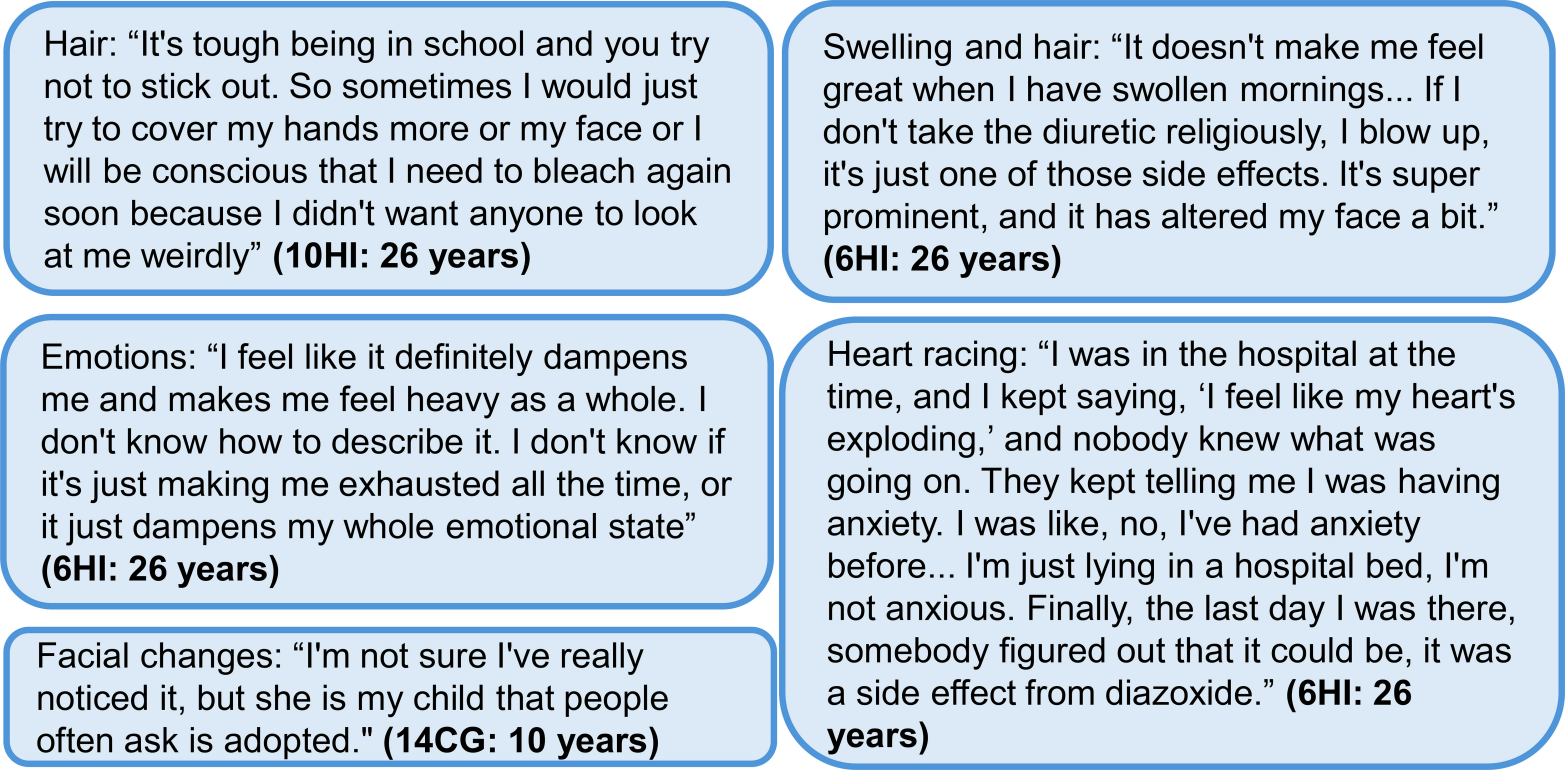
Additional quotes relating to side effects of diazoxide. Participant identifier and age are given in parentheses. In the participant identifier, CG and HI refer to caregiver and person with HI, respectively. HI, congenital hyperinsulinism.

For some participants, one of the more concerning side effects was swelling, which was experienced by 22% of the 121 HIGR participants currently taking diazoxide (**Table 5**). Many participants described their child or themselves as looking puffy or swollen (**Figure 3**). Although this was most visible in their face, it raised concerns about the impact of fluid retention on other body systems that could not be visually identified.

When she went for her G-Tube surgery, she did have a lot of swelling. I had lots of questions then. Is that because of the diazoxide? Is it because of the other medications? My biggest questions were about her heart. I was very scared that she’d get this heart problem, and I wouldn’t notice or (it) wouldn’t be picked up. (9CG: 2 years)

One of the adult participants was especially focused on how fluid retention impacted her self-image and the lengths she went to mitigate this side effect, including taking dandelion root and a diuretic and undergoing lymphatic drainage massage (**Figure 3**). Of the 15 people whose swelling was described in the interviews, 11 had used a diuretic at some point, but a few had discontinued use as they got older.

#### 3.3.3 Side effect: facial changes

Of the 15 individuals discussed during the interviews, 10 commented about diazoxide-related facial changes or features. Of the 121 HIGR participants taking diazoxide, 23% reported facial changes (**Table 5**). These were often discussed by comparing the child with HI with their other siblings or noting similarities between the person and others on diazoxide in the HI community (**Figure 3**).

His whole face just changed compared to even pictures that I had when he was a baby. I don’t even know how to explain it. I looked at other kids that were on the CHI website on Facebook, and it’s like, ‘oh, my gosh, that looks like it could be a sibling of [child].’ Compared to even his real siblings. (3CG: 12 years)

In addition to their general curiosity, two caregivers cited it as the most concerning side effect.

Honestly, the facial features is the one that I think about the most, because it really, sort of indicates how incredibly powerful the drug is if it can actually alter the way that you look. So that scares me, because if it can do that, then what else is it doing? What is long-term exposure to it potentially going to do to her down the road? (13CG: 2 years)

#### 3.3.4 Additional side effects

In the interviews, additional side effects such as dry skin, eczema and skin darkening were considered, but specific attribution to diazoxide did not carry certainty. Of the 121 HIGR participants currently taking diazoxide, 11% reported skin rash as a side effect (**Table 5**). In the interviews, two individuals indicated that their child broke out into a rash when they first started taking the medication, and one of them said that their child still deals with occasional rashes. Another individual discussed a long duration of unexplained pain that has lessened but not fully resolved. Another individual indicated they had a short-term thrombocytopenia problem that resolved. One of the adults with HI articulated additional effects of the medication on her emotional state (**Figure 3**).

#### 3.3.5 Dose impact and timing

When interviewed, individuals reported that the side effects appeared within a few weeks or, at most, a few months after starting diazoxide.

It gradually just ramped up. It wasn’t something that, oh, he took it, and then all of a sudden, he had all this hair, and everything changed. It just was a progression. (3CG: 12 years)

When asked in the interview whether they noticed a difference between the diazoxide dose and the side effects, most people indicated that side effects subsided over time or as the dose decreased; this correlation was especially noteworthy for the volume of hair growth and for swelling.

I think the dose increasing does start the real hair growth … there was a period when we increased her dose, and that hair grew thick and dark on her body. (11CG: 6 years)

However, some participants noted that it was hard to tell if there was a significant difference in the side effects over time or based on dose. One of the parents who had discontinued the medicine described the changes she saw in her child after he stopped taking the medicine.

The water retention, the puffiness of his face, and everything’s not there anymore. I don’t really know about the (facial) structure, I don’t know if I’ll ever know if that really changed or not. The hair growth definitely went away … It was kind of weird seeing him like, oh, wait, that’s how you’re supposed to look. (3CG: 12 years)

#### 3.3.6 Ongoing monitoring of HI and the impact of diazoxide

Families described variable approaches by their clinician for ongoing management to track glycemic control and side effects. Everyone described glucose monitoring as a consistent component of their ongoing care; however, the frequency of these check-ins with their clinician was highly variable based on the age of the person with HI and their center of care. In HIGR, of the 98 caregivers, 72% reported that the participant saw a healthcare professional every four to six months. In the newborn period, individuals were more likely to have monthly check-ins with their clinicians, and check-in frequency then shifted to less frequent as the person grew older and there was a more stable routine for HI management.

There are no tests they didn’t do with me growing up. I was a baby, I had an MRI and a CAT scan, and I also had the neurologist examine me… (Now they monitor me) verbally every six to 12 months? And they ask me how I’m doing and how many episodes I’ve had, and it’s like a 10–20 minute conversation, and that’s it. (10HI: 26 years)

When asked about other ongoing monitoring and tests, individuals mentioned tracking levels of potassium, sodium, and liver enzymes as well as full blood testing, including measuring uric acid, urea, and glycohemoglobin (HbA1c). A few interview participants specified that certain tests were specific to other treatments that they were receiving (e.g., a diuretic or hormones). Two caregivers whose children were aged one and five years mentioned that their child’s doctors still monitored their child’s heart, while the other participants did not. Individuals also mentioned interactions with professionals in other specialties, including cardiology, hematology, neurology, and oncology.

Two caregivers discussed concerns about diazoxide stimulating puberty, and another, how puberty would impact their child’s HI. Often, these concerns were triggered by the appearance of pubic hair and were monitored by their child’s doctor to confirm that there was no sign of precocious puberty.

**Figure 4:**
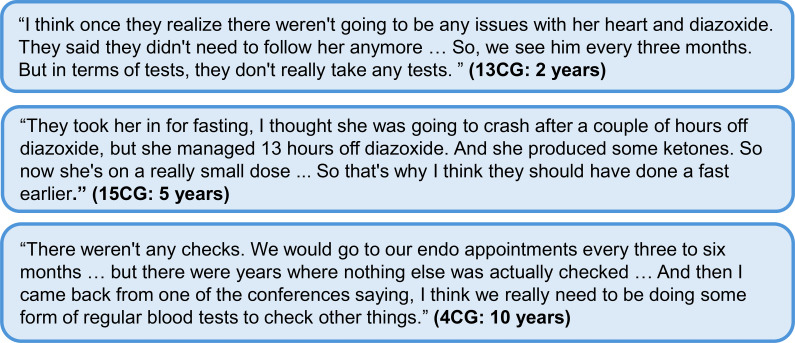
Additional quotes relating to ongoing monitoring of HI and the impact of diazoxide. Participant identifier and age are given in parentheses. In the participant identifier, CG and HI refer to caregiver and person with HI, respectively. HI, congenital hyperinsulinism.

Safety fasts were a prominent subject for seven of the interview participants. Most people found safety fasts helpful in providing a baseline understanding of their child’s fasting tolerance. However, two people stated that the in-hospital fasting environment was stressful and inconsistent with “real life”. Among HIGR participants who had experienced in-hospital fasting (n = 39), only 38% believed the procedure to be consistent with at-home fasting (this is a newly launched question with limited data). The frequency and importance of clinical safety fasts varied between participants (**Figure 4**).

A few parents reported that they had initiated conversations about fasting with their clinicians. Often, this was triggered by conversations with other parents at conferences or on social media, led to a desire for similar safety fasts conducted for their children (**Figure 4**).

I’m just concerned that we don’t have a schedule for these things (monitoring) and the same with fasting, we don’t do any fasting. We don’t; there’s nothing, it’s just, “oh, well, you’re stable enough.” I see other places like [leading world center for HI] where they have a schedule when people go in. I’m torn between I don’t want to be readmitted again, I don’t want to put her through anything unnecessary, but I want to keep her safe. They said that they would be happy to do an echo (cardiogram) every two years, and we could do bloodwork once a year. But I do think it will probably come from me asking for it again rather than from them. (9CG: 2 years)

### 3.4 Glycemic control and individual glucose monitoring

**Figure 5:**
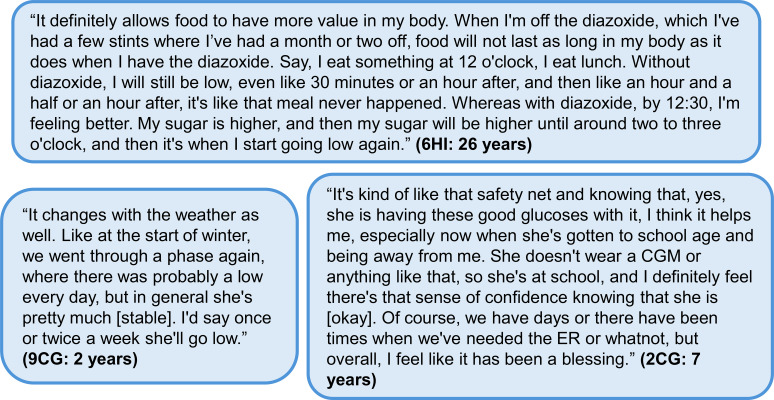
Additional quotes relating to glycemic control and individual glucose monitoring. Participant identifier and age are given in parentheses. In the participant identifier, CG and HI refer to caregiver and person with HI, respectively. CGM, continuous glucose monitoring; ER, emergency room; HI, congenital hyperinsulinism.

When individuals were asked whether they were confident that diazoxide provided them or their child with adequate glycemic control, diazoxide was noted as an important component, but not a stand-alone solution. The largest benefit from diazoxide therapy was perceived as the ability of the family to normalize their routines and have fewer disruptive medical events (**Figure 5**).

I’m confident that diazoxide has me covered. I don’t do more fingerpricks. The only time in those situations that I will, is when he tells me he feels weird. He’s like, “I don’t feel very good.” “Let’s do a poke, let’s just check out to see what’s going on.” (12CG: 8 years)

Caregivers spoke about how diazoxide helped decrease their anxiety. However, due to unexplained hypoglycemia and intercurrent illnesses that increased the risk of hypoglycemia, they still needed to be vigilant.

I’ve worked my butt off over the years to try and make sure that she doesn’t [go low]; there are lots of processes in place and different things that, I suppose, all contribute to the fact that we try to avoid hypos, obviously. So, the medicine is doing its job, I suppose, but just doesn’t keep those random ones from appearing every now and then. (4CG: 10 years)

The frequency of dysglycemia was reported by 110 HIGR participants currently taking diazoxide. A blood glucose level below 70 mg/dL (3.9 mmol/L) was reported by 37% of these participants as occurring up to several times per week, including 18% of the participants, who experienced this more than once a day. Only 3% of the 110 participants reported no longer experiencing blood glucose levels of below 70 mg/dL (**Table 6**). Although less common than a low blood glucose event, a blood glucose level above 180 mg/dL (10 mmol/L) was experienced at least occasionally by 53% of the 110 HIGR participants currently taking diazoxide, with 24% of these reporting a frequency of this level at least one per month. When asked to describe glycemic trends during the interviews, responses focused on blood glucose ranges or the identifiable patterns that led to increased low blood glucose readings (**Figure 5**).

**Table 6:** Dysglycemia experienced by HIGR participants currently taking diazoxide.

	Participants currently taking diazoxide (n = 110)	
Frequency of hypoglycemia or hyperglycemia, n (%)	Hypoglycemia	Hyperglycemia
More than once a day	20 (18)	0 (0)
Once per day	7 (6)	2 (2)
Several times per week	14 (13)	8 (7)
Once per week	15 (14)	4 (4)
Several times per month	13 (12)	8 (7)
Once per month	7 (6)	4 (4)
Several times per year or infrequently	31 (28)	32 (29)
Never	3 (3)	45 (41)
Unknown	0 (0)	7 (6)

HIGR, HI Global Registry.

Hypoglycemia: blood sugar level below 70 mg/dL (3.9 mmol/L, 0.7g/L).

Hyperglycemia: blood sugar level above 180 mg/dL (10 mmol/L, 1.8 g/L).

I tend to get a few hypos, if there’s extra excitement or nervousness or new things, makes it a bit tricky. (4HI: 10 years)

Exercise is probably the biggest thing and I kind of am worried about, as she gets more active, is she going to drop more? (14CG: 10 years)

Exercise, sickness, seasonal patterns, and weather changes were discussed as potential reasons for a decrease in glucose control and an increase in parental anxiety (**Figure 5**). Most participants did not express an expectation that diazoxide would fully address the variations caused by these factors.

For the most part, I think it does its job, but we definitely just have random events. We shouldn’t, in theory, still get hypos if the medicine is doing its job completely. You can never actually just go “here take medicine, you’re gonna be fine, we’re not going to worry about these,” because it just doesn’t happen that way. You can go great for ages, and then it’s like, “bam, here, take this”. Obviously, there are other factors that the medicine doesn’t cover. (4CG: 10 years)

Of all participants in HIGR currently taking diazoxide, 8% were also taking at least one additional medication. Additional medications reported included octreotide, long-acting octreotide, lanreotide, and glucagon. Of those in HIGR currently taking diazoxide (n = 112), 40% reported using at least one food supplement to maintain blood glucose levels. Of the individuals who used a food supplement (n = 45), cornstarch was commonly used (73%).

### 3.5 Impact of disease and medication on daily functioning and quality of life

One common theme among interview participants was the dedication to a consistent schedule and routine. The importance of consistency was most applicable to meals, glucose testing, and medication schedules.

Now I eat very routine-based, I repeat the same things every week in the same order. Very routine, very stick to the program, and at the same time, I always carry snacks with me wherever I go. Food is a very, very big aspect, I would say, of my life. (10HI: 26 years)

Overnight disruptions were discussed frequently, and entailed blood glucose checks, feeds, or general anxiety and fear of hypoglycemia overnight. When 101 caregivers in HIGR were asked if HI rules their life, 61% responded always, very often, or quite often. A few of the caregivers spoke about how their sleep was impacted in the first year of their child’s life, due to the need for overnight care, their own anxiety, and, in one case, their child’s nightly vomiting. Two individuals reported that their child slept in their bedroom longer than the child would have if they did not have HI.

I’m going to say we’re probably up eight to 10 times a night on a good night. They might be little ones, it might be just getting up and repositioning her and then putting the pump back on. This is our bedroom, her crib is at the end of the bed, so we can still hear her and be here for her. I’m way too scared to put her in her own room yet. (9CG: 2 years)

When the 101 caregivers in HIGR were asked how caring for someone with HI affected their decision to have additional children, 67% reported delaying or choosing not to have additional children. Caregivers also reported changes in their own health (**Table 7**).

**Table 7:** Caregiver quality of life report from HIGR by individuals whose children were currently taking diazoxide.

		n (%) of caregivers				
Impact on health	Number of respondents	Not at all	Very little	Somewhat	Quite a lot	Very much
Has the participant’s health in the past 6 months prevented you from taking part in activities you enjoy?	100	17 (17%)	31 (31%)	28 (28%)	18 (18%)	6 (6%)
Do you (as the caregiver of the participant with HI) feel your physical health has suffered from caring for the participant with HI?	101	27 (27%)	24 (24%)	30 (30%)	11 (11%)	9 (9%)
Do you (as the caregiver of the participant with HI) feel your mental health has suffered from caring for the participant with HI?	101	9 (9%)	20 (20%)	28 (28%)	29 (29%)	15 (15%)

Emotional impact	Number of respondents	Never	Seldom	Quite often	Very often	Always
Do you (as the caregiver of the participant with HI) feel that your life is ruled by the participant’s HI?	101	4 (4%)	35 (35%)	22 (22%)	24 (24%)	16 (16%)
Do you feel unhappy because of the participant’s HI?	101	22 (22%)	44 (44%)	18 (18%)	12 (12%)	5 (5%)
Do you worry because of the participant’s HI?	100	0 (0%)	14 (14%)	23 (23%)	22 (22%)	41 (41%)
Do you feel angry because of the participant’s HI?	101	38 (38%)	41 (41%)	9 (9%)	11 (11%)	2 (2%)

Impact on relationships	Number of respondents	No impact	Much stronger	Somewhat stronger	Negative impact	Ended
In general, how would you best describe the impact of the participant’s HI on your relationship with your partner (if you have one)?	91	18 (20%)	21 (23%)	27 (30%)	20 (22%)	5 (5%)
In general, how would you best describe the impact of the participant’s HI on your relationships with family members?	99	42 (42%)	13 (13%)	27 (27%)	17 (17%)	0 (0%)
In general, how would you best describe the impact of the participant’s HI on your relationships with friends?	99	3 (3%)	12 (12%)	52 (53%)	24 (24%)	8 (8%)

HI, congenital hyperinsulinism; HIGR, HI Global Registry. Individuals could choose to answer individual questions and surveys, resulting in a variable number of participants (n) for each question.

**Figure 6:**
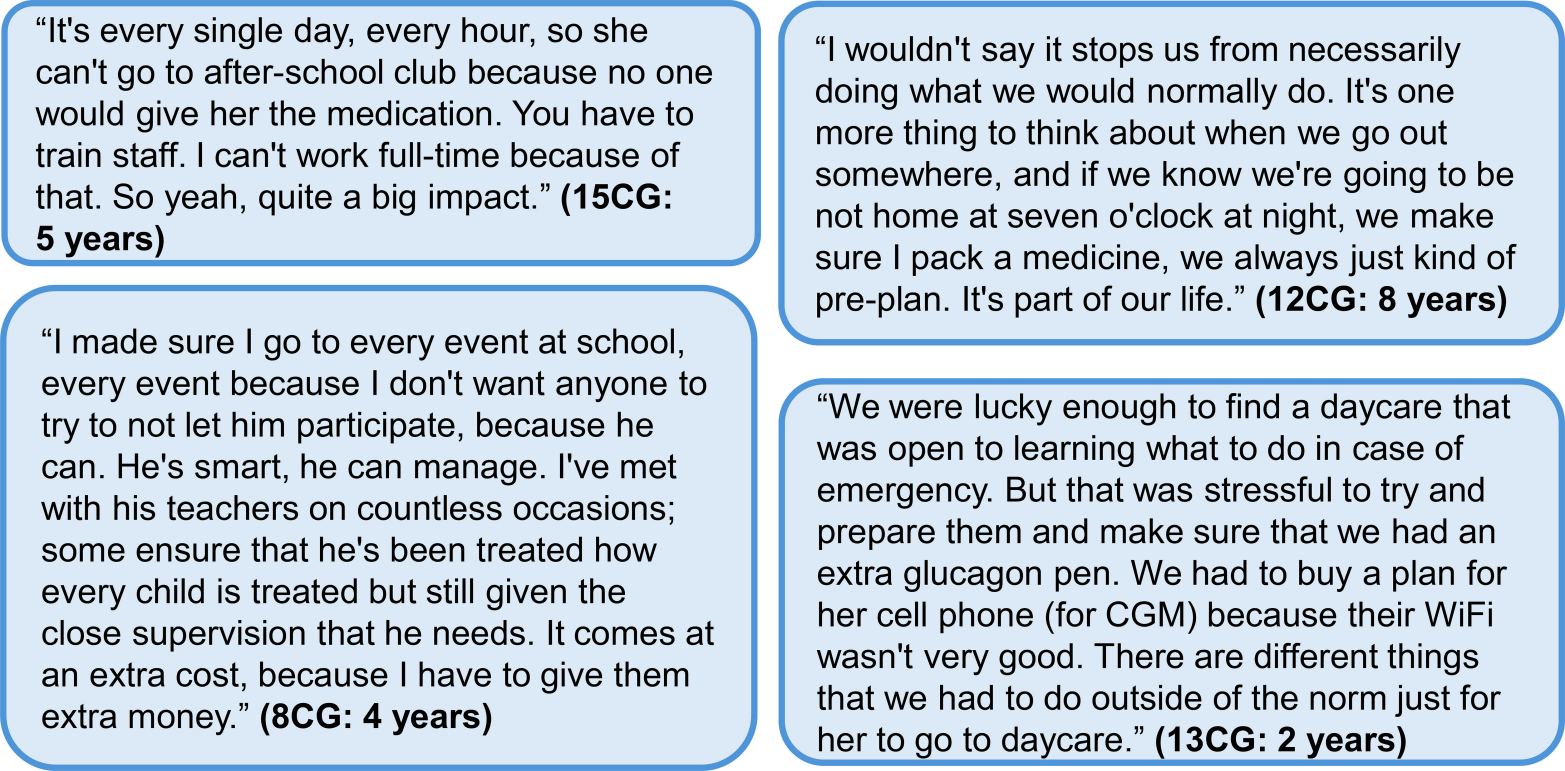
Additional quotes relating to the impact of disease and medication on daily function and the impact at school and daycare. Participant identifier and age are given in parentheses. In the participant identifier, CG and HI refer to caregiver and person with HI, respectively. CGM, continuous glucose monitoring; HI, congenital hyperinsulinism.

One significant impact on day-to-day life was parents’ feeling that they could safely trust others to watch or care for their child; this also impacted the parents’ social lives overall (**Figure 6**). When 24 caregivers were recently asked in HIGR whether they feel comfortable allowing others to care for their child (i.e. babysitting), 71% responded “seldom” or “never.” In some interviews, this discomfort was described as being motivated by the caregiver’s fear or a lack of trust due to the complexity of care needs. However, people indicated that they became more comfortable over time as their child was able to communicate more, the family established routines to better manage the child’s HI, and the child was more compliant with taking their medication. Over time, parents also identified care partners they could trust, including grandparents and specially trained babysitters. Three people noted that they had found nurses or nursing students who were able to serve as part-time babysitters and who the caregivers were comfortable leaving their child with.

It probably wasn’t until he was five, six years old, where I felt more comfortable with a babysitter. Where I think he could tell us more of his needs- if he was hungry. And then even with the babysitter we did have, she was a nursing student, so I felt very comfortable with her because she knew what to look for and she knew how to do a glucose meter. (3CG: 12 years)

#### 3.5.1 Diazoxide and HI at daycare and school

Individuals expressed concern about their child with HI in daycare and school settings (**Table 8**). Some individuals expressed that their school was supportive, especially during mealtimes or with glucose checks. However, others reported that the school was unwilling to support their child’s needs, and that they were told they must be available to pick the child up if they had a low glucose reading, or that they must be present for special events or school trips (**Figure 6**).

**Table 8:** Caregiver quality of life report from HIGR by individuals whose children were currently taking diazoxide.

		n (%) of caregivers					
Impact on participant’s education	Number of respondents	N/A^a^	Not at all	Very little	Somewhat	Quite a lot	Very much
When you were making arrangements for your child to start school, did the school work with you to meet your child’s HI-related needs?	92	36 (39%)	3 (3%)	8 (9%)	14 (15%)	13 (14%)	18 (20%)
How would you rate the participant’s school in meeting his/her health care needs?	90	37 (41%)	13 (14%)	17 (19%)	10 (11%)	6 (7%)	7 (8%)
How would you rate the participant’s school in meeting his/her special education needs?	89	52 (58%)	10 (11%)	11 (12%)	3 (3%)	7 (8%)	6 (7%)

Impact on caregiver career		N/A^b^	None	Rarely	Some	Quite a lot	Very frequently
In the last year, how often have you missed school/work due to the participant’s HI?	99	21% (21%)	13 (13%)	21 (21%)	19 (19%)	15 (15%)	10 (10%)

		N/A	Poor	Fair	Good	Very good	Excellent
How would you rate your employer regarding support to balance your work and the needs associated with the care of the participant with HI (including leaves for health issues, appointments, etc.)?	100	29 (29%)	5 (5%)	8 (8%)	14 (14%)	14 (14%)	30 (30%)

Impact on household			Not at all	Very little	Somewhat	Quite a lot	Very much
Has your household income been negatively impacted due to the participant’s HI?	100		9 (9%)	24 (24%)	28 (28%)	19 (19%)	20 (20%)

			Very poor	Poor	Fair	Good	Very good
How would you rate your ability to pay for the costs associated with caring for the participant with HI?	99		8 (8%)	10 (10%)	20 (20%)	40 (40%)	21 (21%)

HI, congenital hyperinsulinism; HIGR, HI Global Registry; N/A, not applicable. Individuals could choose to answer individual questions and surveys, resulting in a variable number of participants (n) for each question.

a N/A because the participants do not attend school, the majority of individuals in this category are under 6 years of age.

b N/A because the respondent does not work outside of the home.

The children who participated in the interviews were asked if they felt they were treated differently by the other children at school, and they did not think so. However, parents expressed fears of a difference in perception and reported hearing adverse comments from other children or expressed fears of this occurring. Some children also reported that medication or HI management disrupted their school day.

#### 3.5.2 Caregiver career decisions

**Figure 7:**
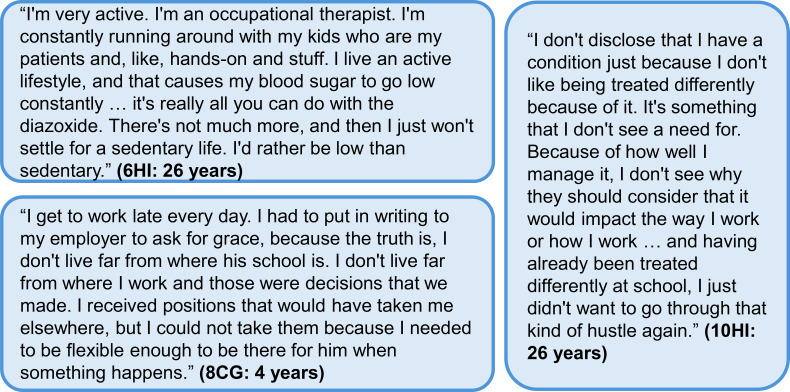
Additional quotes relating to the impact of diazoxide on careers and work. Participant identifier and age are given in parentheses. In the participant identifier, CG and HI refer to caregiver and person with HI, respectively. HI, congenital hyperinsulinism.

In the interviews, all of the career decisions reported within families were specific to changes made by the mother. Many caregivers spoke about either leaving their previous job, limiting their employment, or making career decisions based on needing to be available for care needs (**Figure 7**). In HIGR, caregivers reflected on the impact of HI on their career and household finances (**Table 8**).

#### 3.5.3 Impact on the person with HI

The resilience of individuals with HI was captured in the participant quality of life survey in HIGR. Despite 92% of the 13 participants responding “always,” “very often,” or “quite often” when asked whether they feel as if their life is ruled by their HI, 69% responded that they were able to do everything they want even though they have HI.

The role of diazoxide in this sense of self was especially prominent in reflections of self-image and the impact of glycemic stability on the choices they felt comfortable making for themselves. In the interviews, it was apparent that the interrelationship between HI and treatment was complicated and difficult to separate.

Instead of separating that or this because of medication, I just saw “oh, this is because of the condition” because [if] it wasn’t for the condition, I wouldn’t have to be on medication in the first place … That was my frustration growing up that I had to deal with lots of things that other people of my age and around me couldn’t understand … I do acknowledge that I wouldn’t be half the person I am today. I wouldn’t have the family support, or the family unit that I have. It wouldn’t be as strong as it is, if it wasn’t for its influence. (10HI: 26 years)

Both adult women in the study discussed family planning and the impact their reliance on diazoxide could have on their health and that of their child, especially due to the lack of published data.

### 3.6 Reflections on medication and looking to the future

When people in the interviews were asked to reflect on what improvements they would like to see in treatments for HI, many responded that they wish to understand more about the long-term impacts of diazoxide.

I really want to know more. I don’t just want to be robotized to say “just give it to him, just give it to him”. I just want to know what’s happening on the inside. (8CG: 4 years)

I approached my pregnancy and was going to approach the first few years of my child’s life from a very natural, organic type of thing, and to have that ripped away and understand that she was going to be taking these very potent chemicals on a daily basis, it was hard to accept that. (13CG: 2 years)

Some people were grateful for the medicine and thought that they had found a good routine.

I feel very fortunate that he responded to it. He takes his medication twice a day, morning and night, and at the beginning that seemed very daunting, but then I hear other stories of kids with HI, and in the grand scheme of things, that’s a very easy thing to do is just give him a tiny little dose of medicine in the morning and evening. We feel very fortunate that diazoxide is there and that he was responsive to it. (1CG: 1 year)

Others were motivated to participate in HI studies, such as HIGR, or in other aspects of the HI community to try to advocate for their child or drive research for better care and treatment options. Three people spoke about their interest in participating in clinical trials or finding alternatives to diazoxide. Overall, everyone had the same goal for the person with HI: better glycemic control so the person with HI could lead a life as uninterrupted as possible.

## 4 Discussion

This is the first mixed-methods study to focus on the experience of diazoxide use as reported by individuals taking the medicine and/or their caregivers. Quantitative data from an international patient registry (HIGR) and qualitative data from interviews were utilized to provide a multidimensional perspective on the experience and impact on the family. While diazoxide is a key first-line oral therapy in the treatment of HI, families reported on a range of side effects and a repugnant taste, raising concerns over long-term safety and adherence. Many patients also reported suboptimal efficacy with recurrent hypoglycemic episodes requiring additional therapy adjuncts such as dietary supplementation and intensive feeding schedules to avoid the medical disruptions associated with hypoglycemia and its consequences.

Families tried to maintain consistency and glycemic stability through use of a rigid regular routine; therefore, illness and other disruptions, increased activity, and social activities introduced anxiety and the potential for additional medical problems. Emergency room visits and hospitalizations presented a constant threat to the families and represented an underlying fear of potential brain damage or death. Hospitalizations were a burden not only to families, but also contribute to higher costs to healthcare systems (42, 43). Individuals who were able to maintain better glycemic control were more willing to accept the taste and the side effects of diazoxide.

The most frequently reported side effects from HIGR are consistent with published evidence (8, 19, 21, 24, 33) as are the smaller proportion reporting more severe side effects, including thrombocytopenia and pulmonary hypertension. Additionally, this study provides context and impact from the caregiver’s perspective, reinforcing the patient/caregiver voice in the management of HI, a complex, long-term condition associated with the risk of neurodisability (3, 4, 18, 25, 32).

While changes in physical appearance are well recognized in patients using diazoxide over prolonged periods (5, 8, 9, 18, 27, 44). This study is the first to acknowledge the social stigma and impact on self-esteem, prompting the need for clinicians to address these concerns and support patients in how they cope. As drug adherence is generally well correlated with side effect profile (45), patient-facing understanding of long-term therapy implications, including psychosocial ramifications, needs to be explored for improved and tolerable treatment outcomes. Although some studies have investigated long-term use (46), parents reported concerns about other unknown aspects of diazoxide, therefore additional studies should be conducted, and better information should be provided to patients to further alleviate their concerns.

A large proportion of HIGR participants currently taking diazoxide (64%) experienced feeding problems previously reported in other studies (46). This study reinforced the context of gastrointestinal adverse impact by interview participants discussing force-feeding, increased anxiety around mealtimes, and vomiting associated with overeating. These narratives have not been reported but need to be understood to plan holistic patient care. This study did not explore long-term dietary patterns and an individual’s relationship with food, but one adult reported their diagnosis of anorexia. The complex relationship between the illness manifestations, treatment effects, and side effects on gastrointestinal manifestations has not been well characterized; our study findings provide insight into the acceptance and adaptations of imperfect therapy, indicating the need to explore the social perspectives for new therapies in development. Additionally, new therapies should strive to eliminate the need for supplemental nutrition to maintain euglycemia and reduce feeding issues and gastrointestinal distress.

An important angle, rarely reported in published evidence, is the caregiver burden (mainly affecting mothers), which can cause anxiety and stress, often due to the need to adhere to stringent routines. Mothers reported on disrupted sleep, their added stress of participating in social activities, and the other ways HI impacted their parenting methods. The finding is not surprising, considering that mothers assume greater responsibility for managing chronic illness (47–50). Further, 93% of people populating HIGR were mothers and only mothers participated in qualitative interviews. Interestingly, the children in the study did not report experiencing the stress and anxiety their mothers did.

In contrast, adults with HI were aware of how HI and diazoxide shaped their life experiences. There was resolve and resilience that seemed rooted in the desire to escape the necessary over-protective environments they grew up in. Building safety nets that were not overly restrictive and advocating for their needs with partners, friends, and other people in their lives was discussed as critically important. These findings indicate the need to integrate multidimensional changeable perspectives to optimize therapy outcomes.

### 4.1 Limitations

Individuals living with HI and caregivers volunteered to participate in this study; this may have inadvertently contributed to a selection bias in both the qualitative and quantitative components, including what individuals decided to share. As HIGR is an electronic registry, caregivers less comfortable with technology may have opted out. Further, interviews were conducted in English. Although the study team made reasonable efforts to widen recruitment for patients in different centers and countries, given the rarity of HI, it was not possible to stratify participants for these variables.

As a cross-sectional study, individuals were not followed over time, but the qualitative interviews were designed to include viewpoints at different phases of life. However, the sample size in each age category was small, precluding meaningful longitudinal narratives. It is possible that additional themes might have emerged with interviews over a wider age span. However, the conjunction of registry review and qualitative interview provides reasonable robustness to our data. Nonetheless, the study team recognizes the need to widen interview participation from different countries to capture the impact in different family, social, cultural, and economic settings. This is particularly important as diazoxide is deemed an essential medicine by the World Health Organization, but access is still limited or unavailable in many countries (9, 18, 32, 36). Geopolitical factors including healthcare affordability and overall access may further impact the generalizability of the results. However, through the advocacy and research work of CHI (32, 36), the authors are aware of ongoing access issues and are working on expanding participation within these communities, including by expanding the number of languages.

Caregiver responses in HIGR may have been subject to recall bias. However, caregivers and patients living with a rare disease are often highly engaged in managing the condition and often keep extensive records (51–53); therefore, it is unlikely that HIGR responses were significantly influenced by recall bias. Recruitment and retention are common problems in rare disease registries. To address these challenges, CHI conducts continuous engagement for HIGR.

Due to the heterogeneity of HI phenotypes, the study results were not stratified by objective characteristics such as glycemic control, drug dosage, or the outcomes of fasting tolerance tests. However, such stratification is anchored in a clinician perspective. By contrast, our study is patient-focused and has generated complementary information about the feelings and observations of the use of diazoxide in the context of daily life in families managing HI.

It is possible that HIGR questionnaires were misinterpreted, leading to incorrect responses; this is mitigated through implemented data validation, quality standards, and communications practices to monitor data reliability. At present, the quality of life tool within HIGR remains unvalidated, in the absence of a disease-specific instrument. It is possible that perceptions around quality of life were not fully representative; however, HIGR quality of life domains are well aligned to responses derived from quantitative and qualitative sections of the study. Further, patient feedback on the use of the quality of life tool suggests wide user acceptance, supporting a future validation study.

### 4.2 Future directions

The interview discussions raised additional themes in the use of diazoxide therapy that require future study; these include access to treatment, real-world continuous glucose monitoring application, management of feeding problems, family planning, and the impact on siblings and other family members. As the sample size increases, future studies should include more subgroup analyses for stratification by additional characteristics such as dosage, length of use, and genetic variants to further understand related side effects and glycemic control. Another important area of future research is the long-term impact of the fear of hypoglycemia from the limits of diazoxide efficacy on some caregivers’ mental health.

The results of this study have implications for medical professionals and families living with HI. To optimize diazoxide efficacy and minimize side effects, the study suggests the need for greater adoption of the International HI Care Guidelines and for stronger dialogue between families and professionals (8). The study also points to the considerable psychosocial impact, both for the parents and children living with HI, with implications for monitoring and treating mental health, the need for support structures, and awareness of changing perspectives between childhood and adolescence with long-term use of diazoxide. Within the study, some individuals spoke about the value of connecting with other families, but more family-oriented resources can be developed and disseminated to schools and other caretakers. The study showed there is a clear unmet need for improved novel therapies for HI. Many individuals with HI and their families are striving for a future where their lives are unburdened by the constant focus on euglycemia, and the inescapable efforts to maintain it, to prevent the negative consequences of HI.

## Data Availability

The raw data supporting the conclusions of this article will be made available by the authors, without undue reservation.

## Supplementary material

Supplementary Table 1 Examples of qualitative codebook and major themes to emerge from qualitative analysis. GI, gastrointestinal; CGM, continuous glucose monitoring; HI, congenital hyperinsulinism.
