# Supplementary material for: Real-world experience with the use of diazoxide among people living with congenital hyperinsulinism and their caregivers
Source: Front Endocrinol (Lausanne). 2025 Aug 21;16:1628125. doi: 10.3389/fendo.2025.1628125 (PMC12410177; doi:10.3389/fendo.2025.1628125)
Supplement: Supplementary Table 1 — Examples of qualitative codebook and major themes to emerge from qualitative analysis. GI, gastrointestinal; CGM, continuous glucose monitoring; HI, congenital hyperinsulinism. [file Table1.docx]

**SUPPLEMENTARY TABLE 1 Examples of qualitative codebook and major themes to emerge from qualitative analysis.**

| **Code name and definition** | **Sub-code examples** | **Open code examples** |
| --- | --- | --- |
| **Taking diazoxide:** Descriptions of the experience of taking diazoxide including how the modality and taste of the drug impact the experience | Modality | Syringe  Liquid  Tablets |
|  | How taken | Coax  Tantrum  Just does it |
|  | Taste | Bitter  Sour  Disgusting |
| **Side effects**: Descriptions of diazoxide-related side effects, including observations on the onset, management of the side effects, and related concerns | Concerns about side effects | Fear what I cannot see  Gnarly  Don’t fully understand |
|  | Fluid retention | Puffy  Swollen |
|  | Hair | Fuzzy  Hairy |
|  | Other side effects | Palpitations  Dampens emotional state  Gum bleeds |
| **Impact of diazoxide and HI on diet, feeding, and GI health:**  Descriptions of how HI and diazoxide impacted diet, feeding, and GI health | Gastrointestinal (GI) issues | Reflux  Stomach swollen  Vomiting |
|  | Feeding considerations | Cater  Forcing  Same every day |
|  | Feeding issues | Swallowing  Refusal to eat  Feeding aversion |
|  | Levels of appetite | Suppressed appetite  Stimulated appetite  Lost appetite |
| **HI management and glycemic control:** Descriptions of HI management, including glucose monitoring, diazoxide use patterns, and subsequent glycemic control | Glycemic control | Prevents lows  Raises blood sugar  Sugars |
|  | Hypoglycemia | Episodes  Crashes  Lows |
|  | Dose | Grew out of  Held doses  Weaned  Increased dose |
|  | Glucose monitoring | Pokes  Blood sugar checks  Finger pricks  CGM |

GI, gastrointestinal; CGM, continuous glucose monitoring; HI, congenital hyperinsulinism.
